# Observations on Curvatures of the Spine

**Published:** 1824-11

**Authors:** James Macartney

**Affiliations:** Professor of Anatomy and Surgery in the University of Dublin, &c. &c.


THE LONDON
### Medical and Physical Journal.

No 5 OF VOL. LII.] NOVEMBER, 1824. [No 309.

For many fortunate discoveries in medicine, and for the detection of numerous errors, the world is indebted to the rapid circulation of Monthly Journals; and there never existed any work, to which the Faculty, in Europe and America, were under deeper obligations, than to the Medical and Physical Journal of London, now forming a long, but an invaluable, series.—RUSH.

### ORIGINAL COMMUNICATIONS,
### SELECT OBSERVATIONS, &c.

ART. I.—*Observations on Curvatures of the Spine.* By JAMES MACARTNEY, M.D. M.R.I.A. F.R.S. Professor of Anatomy and Surgery in the University of Dublin, &c. &c.

*To the Editors of the London Medical and Physical Journal.*

GENTLEMEN,—The paper I send you was printed in the Transactions of the Royal Irish Academy, in the year 1817; but, as none of the late writers on Curvatures of the Spine have noticed this Essay, I shall be obliged by your giving it greater publicity than it seems to have yet possessed, by inserting it in your Journal; and am your very obedient servant,

JAMES MACARTNEY.

DISTORTION of the spinal column may arise from three causes. The first is that peculiar disease, which, terminating in the ulceration of the bodies of the vertebræ, necessarily induces an abrupt and very evident curvature; or, more properly, an angular figure of the spine. The second is that state of the bones, in which, from a deficiency of earthy matter in their composition, they are incapable of preserving their natural form; but bend or diminish under the operation of pressure. The spine, in those circumstances, will exhibit very different degrees of curvature, in proportion to the softness of the bones. In some persons, the deformity may be very slight; but, in others, there may be several very considerable lateral flexures of the spine. The third cause of distorted spine is the feebleness of those muscles which are employed in maintaining the erect position of the body. The tendency of a weakened state of the muscles to augment the more serious kinds of curvature, and to induce, even without a softened condition of the bones, an evident distortion of the back, does not appear to have yet received sufficient consideration, either from medical men, or from those whose interest it is early to notice, and prevent, deformity in young people.

Slight curvatures are too often neglected; and hence, the wrong direction being given to the spinal column of bones, a serious and permanent distortion is apt to be subsequently established during a period of ill health, or by confinement to some one attitude from peculiar studies or occupation.

Slight curvatures of the spine are sometimes also disregarded, under the erroneous supposition that they will afterwards be concealed by modes of dress. No means, however artful or ingenious, will serve to disguise any distortion of the spine so well, that its injurious effects on the whole figure will not be felt, although the cause of the disagreeable appearance may not be known. A perfectly straight spine communicates to the motions, not only of the trunk, but of the extremities, a facility and grace which secretly and irresistibly influence our judgment of the figure, in the same manner as the countenance, sooner or later, determines our opinion of the face.

Spinal curvature is so much more frequent in females than men, than it might, with great reason, be considered as a complaint peculiar to the sex.

These circumstances render it highly necessary to watch and prevent every deviation from the natural form of the back in growing girls.

The progress of spinal curvature, where the other bones do not exhibit an undue flexure from softness, is very insidious. Distortion may exist for a considerable time, without being known. Parents are usually the last to notice it. Before there is any perceptible deformity, the young person may be observed to avoid, when at rest, the erect position of the body, by reclining on her companions, or against any near object; or will be seen to prefer some particular position, in which the weight of the head is thrown off the middle line of the body. In leaning, also, to write or draw, one side will be often favoured more than the other; and no admonition will be sufficient to check the disposition to seek rest for the spine.

The first appearance of deformity is usually an unequal projection of the shoulder-blades, which is supposed to be a defect in the situation of the scapula itself. Every degree of lateral curvature in the spine causes a corresponding prominence of the ribs, on the side to which the vertebræ are bent; and this again produces the eminence of the shoulder-blade on the same side.

In examining the external appearance of the spine, the person should be made to sit, for some time, with the body erect, in order to produce a degree of fatigue; when, if there be any tendency to lateral curvature, it will be detected by the spine resting itself by inclining towards the side, rather than forwards. The degree of curvature may be ascertained by tracing the projection of the spinous processes along the back, the situation of each of which should be marked with ink for future instruction.

In place of the lateral curvature, it sometimes happens that the natural S-like flexures of the spine are increased, so as to produce a degree of deformity. This is particularly to be observed with respect to the dorsal flexure of the spine, in children of weakly constitutions, whether male or female. The stooping of the shoulders, which marks the approach of old age, is also to be accounted for by the diminished strength of the muscles of the back. I have known but two cases, in which the natural backward flexure of the loins was increased so as to amount to distortion.

When curvature of the spine has existed for any length of time, the bodies of the vertebræ which are concerned conform to the wrong inclination the spine has assumed. They are rendered thin, on one side, by absorption; whilst the other side preserves, or in some places exceeds, its natural thickness. When any considerable curvature has existed for a length of time, it necessarily induces a second in an opposite direction of some other part of the spine; and this again often gives rise to a third, in the direction of the first.

The means which are most commonly supposed to be necessary for avoiding deformities of the spine, are, I believe, those which have the greatest share in producing the usual spinal curvature. I allude to the attempts which are made to oblige young people to maintain the body immoveably in the erect posture; and to the restraint which is imposed on the development of the trunk, and the exercise of its muscles, by the early use of stays.

It may not be improper to state some physiological principles, on which the above opinion is founded.

It is a well-known law of muscular action, that it cannot be maintained without intervals of repose. No determination will enable a person to hold the arm, in the extended position, more than a few minutes. The sensation of fatigue is more intolerable than the greatest pain. We have frequent opportunities of seeing this exemplified during surgical operations.

It is generally acknowledged, that muscles are strengthened by being exercised; but the difference between the ordinary actions of muscles, and those attended with effort, has been overlooked. The bulk and strength of muscles are not increased by the customary actions, although ever so often repeated; while exertion of muscles beyond their habitual operations, even with long intervals of rest, will augment their magnitude and power to an extent that it is difficult to limit. This law of muscular action applies equally to the voluntary and involuntary muscles. The heart, the diaphragm, and the muscles so constantly employed in speech, in the motions of the eyes and lips, gain no additional bulk during life, unless exerted to an unusual degree.

As examples of a different kind, I may mention the enormous bulk the anterior muscles of the thigh arrive at in old stage-dancers, and the great strength the muscular parts of internal organs acquire, when there exists any obstruction to the expulsion of their contents.

A most extraordinary example of the last kind came under my knowledge some months ago, in which the left ventricle of the heart, in consequence of a diseased state of the aortic semilunar valves, had attained a degree of power, by the constant efforts it had to make, so disproportionate to the strength of the arteries, that the smaller branches of the latter were ruptured in several parts of the body, and coagula of blood formed, especially in the substance of the liver, and behind the peritoneum. The tunic of the liver at length gave way, and the patient died, from the quantity of blood that was shed into the cavity of the abdomen.

Another law of muscular action deserves to be noticed. Muscles lose power and bulk by disuse; but what would be disuse in one case, would not constitute it in another; or, in different words, in proportion to the frequency with which a muscle is intended to act, the necessity for its exercise exists, in order to prevent its degeneracy. There are some muscles in the human body, which are rarely put into full action. In animals we have examples of muscles which are only intended to be employed on particular occasions, that may never occur. Such muscles do not decline from disuse; whilst those that are provided for constant employment cannot remain at rest, without sustaining a great diminution of their bulk and power.

Now, if we apply the preceding principles to the muscles attached to the spinal column, the injurious effects of exhausting their strength, by attempting to maintain any one permanent attitude of the body; of restraining their ever-varying motions; or of substituting, for their natural support to the bones of the spine, the artificial one of stays, must be sufficiently obvious.

Experience appears to accord exactly with theory on this subject. We find that those distortions of the back which are in a degree almost universal amongst women of the better ranks of life, rarely occur in the other sex, who do not employ an external support to the spine; that they are equally rare in females who have never worn stays; and that those women who have been early accustomed to carry burthens on the head, are quite remarkable for the straightness of the spine, and correctness of the form of the shoulders.

I might add, that the neck, of which the motions are unconstrained by modes of dress, is scarcely ever distorted; although the weight of the head is chiefly borne by this portion of the spine.

As far as I have had the means of judging, or of collecting information from others, these remarks are equally applicable to the inhabitants of other countries, as to those of Ireland.

Besides the longitudinal muscles which are chiefly employed in erecting the spine, the flat muscles, which execute the rotatory motions of the shoulder-blade, are often not fully developed, from want of being exerted; the consequence of which is, that the weight of the upper extremity constantly tends to bring it downwards and forwards, producing too great a roundness of the shoulders, and a projection or constant elevation from the ribs of the posterior edge of the shoulder-blade.

If it came within the scope of this Paper, I could show many evil effects on the shape of the whole female bust, from external constraint. It is sufficient to state at present, that I have found those women, and only those, who never wore any other than loose vestments, to possess the form and proportions which are displayed in the most beautiful of the antique statues.

In seeking a remedy for distortions of the spine, it is not unnatural to place great reliance on different kinds of machinery. The operation of bandages and of artificial supports would appear, at first, to be very obvious and intelligible. The application, however, of mechanic force or pressure to the living body, with the view of rectifying deformities, requires the greatest care, discretion, and knowledge, of the vital properties of the different tissues engaged. It is too often entrusted to the judgment of mechanics, who construct and sell surgical apparatus, and thereby gain a certain degree of experience, undirected by any principles; a species of knowledge, which is always dangerous in those who have to manage the various and complicated powers of the living system.

It would be departing from the plan of this Paper, to enter into the merits or faults of various mechanical contrivances, which have been devised for correcting deformities of the trunk. The principle, however, on which they should be alone constructed and applied, may be stated to be that of removing weight and pressure from parts, which could not be enabled, by other means, to sustain the operation of these mechanic powers. All instruments have injurious effects, as far as they limit or interrupt the natural actions of the muscles. It is by muscular action that we not only move, but sustain, any one position of the body. The application of a splint to a broken leg is unavoidable; but, if the instrument were continued to be worn, it would ultimately render the limb useless, by destroying the power of its muscles. Nature, indeed, sometimes adds elastic power to muscular, for the purpose of aiding and relieving the latter. This advantage is provided by the elastic ligaments of the spine in the human body, and still more so by those of the heavy-headed quadrupeds. If such a combination of power could be imitated by art, it would, no doubt, prove highly useful; but nothing of the kind has yet been attempted.

Mechanic motion and vital action are so different in their laws and nature, that it becomes necessary to make the former subordinate to the latter in the living body. I recollect but one instance in the structure of animals, where nature has substituted the one for the other: it is the joint of the tibia with the metatarsus in the stork, in which there is a mechanical contrivance for allowing the animal to rest, while standing on one leg, without any muscular effort.*

External machinery cannot be affixed to the body without in itself producing pressure somewhere. This may be well illustrated by the use of monitors and bandages for drawing the shoulders back, and, as it is ignorantly supposed, for opening the chest.

When these instruments leave any permanent effect, it is a hurtful one. The muscles of the scapula are rendered weak by disuse; the convexity of the superior ribs, on which the breadth and expansion of the chest depend, is diminished; and the anterior part of the ribs and sternum are thrown forwards, causing that shape which is known under the name of *hen-breasted*.

I have known several instances in which worse consequences followed this lateral compression of the chest in young women; such as difficult breathing and altered function of the skin, impaired powers of digestion, and great emaciation, in persons who had previously enjoyed robust health.

When it becomes necessary to relieve the spine from the weight of the superior parts of the body and the head, until by proper medical treatment the strength be sufficiently restored, I prefer the mode of resting on a flat surface, or, what is more comfortable, a gently-inclined plane, to any species of machine or bandage that has yet been invented. I think, however, that the effects of the recumbent posture are not always understood, and are sometimes over-rated. There appears to be no necessity for enjoining rest, as is usually done. On the contrary, the patient should rather be encouraged to exercise the spinal muscles by rolling about, and moving the arms and legs at pleasure; for which purpose a large platform, stuffed with wool, or a mattress, on which children can have their toys and plays with

* Dumeril and Cuvier have noticed this mechanism, but have unaccountably described it as existing in the joint of the knee.

their companions, is preferable to a sofa or plain board, which are most frequently employed.*

The recumbent position is sometimes resorted to as the sole means of curing, or of preventing, curved spine. In several celebrated boarding-schools in London, it is used indiscriminately for all species and degrees of distortion; and, in some seminaries, all the girls are condemned to get their lessons, during so many hours every day, in the supine posture, with the view of preventing the occurrence of deformity. It appears to me more than unnecessary to confine healthy girls to the recumbent position; yet too much caution cannot be used to prevent children leaning uniformly to one side, in the act of writing, drawing, &c.—more especially if they be weakly, or if they seem to prefer that particular position.†

The advantages from exercising the spinal muscles in various positions of the body, should never be lost sight of; both as the means of preventing and of curing curvature, when the erect position can be safely sustained. In some cases, where the curvature has been slight, the persons very young, and the general health good, I have succeeded in restoring the natural form, merely by liberating the body from all constraint of stays or monitors, and permitting as much exercise to be taken as could be borne without fatigue.

Balancing any object, however light, on the head, has great efficacy in rectifying the shape of the spine. It is also readily adopted, as a pastime, by young people. In some instances of spinal curvature, where there was not a softened state of the bones, I have tried, with the best effects, exerting the muscles of the back, by the carriage of a bag of sand on the head; employing, at first, short periods of time for the exercise, and allowing rest in the intervals, in the recumbent posture; and, as the strength increased, repeating the exertion more frequently, and continuing it longer.

I have every reason to believe that this treatment, if employed with proper caution, will be always useful in cases of curvature from weakness; and, if practised with assiduity during early

---

* I do not apply these observations to curvature from carious or ulcerated vertebræ, nor do I intend to enter into the consideration of that disease at present; there being no difference of opinion, with medical men, respecting the propriety of treating it by rest and external stimulation.

† It occasionally happens that strong and labouring people acquire some lateral inclination of the body, and a projection of the shoulder-blade, in consequence of employing one hand almost exclusively in their work. I have observed, in these cases, that the shoulder which is the least exercised, is the one that stands out, whilst the other is apt to be peculiarly well formed; a fact which corroborates the principles laid down in the preceding part of this Paper, and suggests the propriety of teaching young people, as much as possible, to become ambidextrous.

youth, I am persuaded would be the best means of preventing distortion.

The means which are most beneficial where merely the shoulders are round and prominent, are skipping with a rope, and the use of the dumb-bells; particularly in the action of carrying them backwards, which exerts the flat muscles that lie between the shoulder-blades and the spine.

I shall conclude by observing, that in all cases where a curvature of the spine, from a softened state of the bones, is apprehended or has taken place, every endeavour must be made to improve the health and strength, by a purer air, tonic medicines, and the cold bath; without which, machinery and rest will be useless, and exercise hurtful.

Some situations are much more productive of a disposition to curved spine than others. In certain districts of England and Holland, it is found to prevail. In the neighbourhood of Leyden, I observed great deformity of the spine to be almost universal with the women, and to exist even frequently in men. Damp and low situations would seem to have a peculiar tendency to induce a softened state of the bones.

With respect to medicines, it is generally known that preparations of iron are the best tonics in this disease. In the employment of the cold bath, I have found great advantage from having it used in the manner of affusion; not only in cases of weak spine, but whenever the object was to restore strength, and increase the activity of the circulation. I have also employed the affusion of tepid water, with very marked benefit, in cases where, either on account of the season or other circumstances, the dashing of cold water over the body would have produced too great a shock.

If the person be young, and the curvature not of very long standing, although it may have produced great deformity, there is no reason to despair of a perfect recovery. As long as the growth of the body is going on, there is a considerable tendency in all the parts to return to their destined form and uses, when placed in proper circumstances, and when the actions of the vascular system are vigorous and healthy. Thus we find even fractured and dislocated bones make great advances, in young people, to their original figure or situation. The gradual reduction and shaping of the osseous case in necrosis, also belong to the same law of the animal economy.

